# The Additional Contribution of White Matter Hyperintensity Location to Post-stroke Cognitive Impairment: Insights From a Multiple-Lesion Symptom Mapping Study

**DOI:** 10.3389/fnins.2018.00290

**Published:** 2018-05-01

**Authors:** Lei Zhao, Adrian Wong, Yishan Luo, Wenyan Liu, Winnie W. C. Chu, Jill M. Abrigo, Ryan K. L. Lee, Vincent Mok, Lin Shi

**Affiliations:** ^1^Department of Medicine and Therapeutics, The Chinese University of Hong Kong, Shatin, Hong Kong; ^2^Department of Imaging and Interventional Radiology, The Chinese University of Hong Kong, Shatin, Hong Kong; ^3^Chow Yuk Ho Technology Centre for Innovative Medicine, The Chinese University of Hong Kong, Shatin, Hong Kong; ^4^Therese Pei Fong Chow Research Centre for Prevention of Dementia, The Chinese University of Hong Kong, Shatin, Hong Kong; ^5^Lui Che Woo Institute of Innovative Medicine, The Chinese University of Hong Kong, Shatin, Hong Kong; ^6^BrainNow Medical Technology Limited, Hong Kong Science and Technology Park, Shatin, Hong Kong

**Keywords:** lesion location, white matter hyperintensity, cognitive impairment, ischemic stroke, multiple-lesion symptom mapping, support vector regression

## Abstract

White matter hyperintensities (WMH) are common in acute ischemic stroke patients. Although WMH volume has been reported to influence post-stroke cognition, it is still not clear whether WMH location, independent of acute ischemic lesion (AIL) volume and location, contributes to cognitive impairment after stroke. Here, we proposed a multiple-lesion symptom mapping model that considers both the presence of WMH and AIL to measure the additional contribution of WMH locations to post-stroke cognitive impairment. Seventy-six first-ever stroke patients with AILs in the left hemisphere were examined by Montreal Cognitive Assessment (MoCA) at baseline and 1 year after stroke. The association between the location of AIL and WMH and global cognition was investigated by a multiple-lesion symptom mapping (MLSM) model based on support vector regression (SVR). To explore the relative merits of MLSM over the existing lesion-symptom mapping approaches with only AIL considered (mass-univariate VLSM and SVR-LSM), we measured the contribution of the significant AIL and/or WMH clusters from these models to post-stroke cognitive impairment. In addition, we compared the significant WMH locations identified by the optimal SVR-MLSM model for cognitive impairment at baseline and 1 year post stroke. The identified strategic locations of WMH significantly contributed to the prediction of MoCA at baseline (short-term) and 1 year (long-term) after stroke independent of the strategic locations of AIL. The significant clusters of WMH for short-term and long-term post-stroke cognitive impairment were mainly in the corpus callosum, corona radiata, and posterior thalamic radiation. We noted that in some regions, the AIL clusters that were significant for short-term outcome were no longer significant for long-term outcome, and interestingly more WMH clusters in these regions became significant for long-term outcome compared to short-term outcome. This indicated that there are some regions where local WMH burden has larger impact than AIL burden on the long-term post-stroke cognitive impairment. In consequence, SVR-MLSM was effective in identifying the WMH locations that have additional impact on post-stroke cognition on top of AIL locations. Such a method can also be applied to other lesion-behavior studies where multiple types of lesions may have potential contributions to a specific behavior.

## Introduction

Preexisting white matter hyperintensity (WMH) is frequent in ischemic stroke patients. Recently, the global and regional WMH burden has been realized as an independent risk factor for cognitive impairment early after stroke. The total WMH volume predicted poor cognitive performance after stroke independent of acute infarct volume in minor (Sivakumar et al., 2017; Zamboni et al., 2017) and mild-to-moderate (Kliper et al., 2014) stroke, especially in processing speed (Jokinen et al., 2005; Prins and Scheltens, 2015) and executive function (Wen et al., 2004; Jokinen et al., 2005; Prins and Scheltens, 2015). In terms of post-stroke outcome prediction, the WMH lesion burden also influenced the critical outcome-predicting infarct thresholds (Patti et al., 2016). In addition, several studies investigated the impact of WMH location on post-stroke cognitive impairment, and found that periventricular WMH (Prins et al., 2004; Jokinen et al., 2005; Kang et al., 2013) or deep WMH (Kandiah et al., 2011; Kang et al., 2013) were associated with cognitive deficits and incident dementia after stroke independent of the presence of cerebral infarcts. Furthermore, preexisting WMH might also have a potential long-term effect on post-stroke cognition, especially in patients with lacunar infarcts where the post-event cognitive deficits caused by acute lesions were relatively temporary (Kang et al., 2013; Sivakumar et al., 2017). However, most of these studies did not exclude the patients with prior stroke, which is a significant confounder when evaluating the impact of WMH on post-stroke cognition. In addition, it is still not clear whether WMH locations are associated with post-stroke outcomes if infarct locations are also considered. We hypothesized that there are certain strategic WMH locations that have independent contributions to short-term and long-term post-stroke cognitive impairment regardless of infarct volume and locations.

This hypothesis can be validated with lesion-symptom mapping (LSM) analysis, but the existing LSM methods (either mass-univariate or multivariate LSM) still fall short in terms of analytical power and need to be improved. Although multivariate lesion-symptom mapping methods have been realized as superior to mass-univariate lesion symptom mapping in terms of sensitivity and accuracy (Karnath and Smith, 2014), they only considered the presence of a single type of lesion. If two (or more) kinds of lesions generally coincide in the brain (for example acute infarct as a major event and WMH as a secondary event) and are both important for the behavior, there might be some distortions in the expected strategic regions of the major event (e.g., acute infarct) identified by these conventional lesion symptom methods, and the power of behavior prediction by the lesion sites of the secondary event (e.g., WMH) might be underestimated.

In this regard, we aimed to develop a multiple-lesion symptom mapping (MLSM) model that simultaneously considers the presence of different kind of lesions (acute infarct and WMH in this case) on a voxelwise basis. Inspired by the recently developed multivariate lesion symptom mapping approach using support vector regression (SVR-LSM) (Zhang et al., 2014), we proposed a multiple-lesion version, the SVR-MLSM, which considers not only inter-voxel correlations within acute ischemic lesions (AILs) but also inter-lesion correlations between AIL and WMH throughout the brain. The SVR-MLSM model was applied to a first-ever stroke cohort with AILs in the left cerebral to investigate the strategic WMH locations for short-term (3–6 months after stroke) and long-term (15–18 months after stroke) cognitive deficits post stroke. To evaluate the relative merits of SVR-MLSM, we also performed LSM analyses that only considered the presence of AIL (including the mass-univariate VLSM and the SVR-LSM), and compared their behavior prediction performance based on the significant clusters of AIL and/or WMH of these models. Different volume control strategies were attempted in these LSM models for a more comprehensive comparison. In addition, we also compared the significant WMH locations identified by the optimal SVR-MLSM model for the short-term and long-term cognitive impairment after stroke.

## Theory

### MLSM through multiple regression

**Figure 1:**
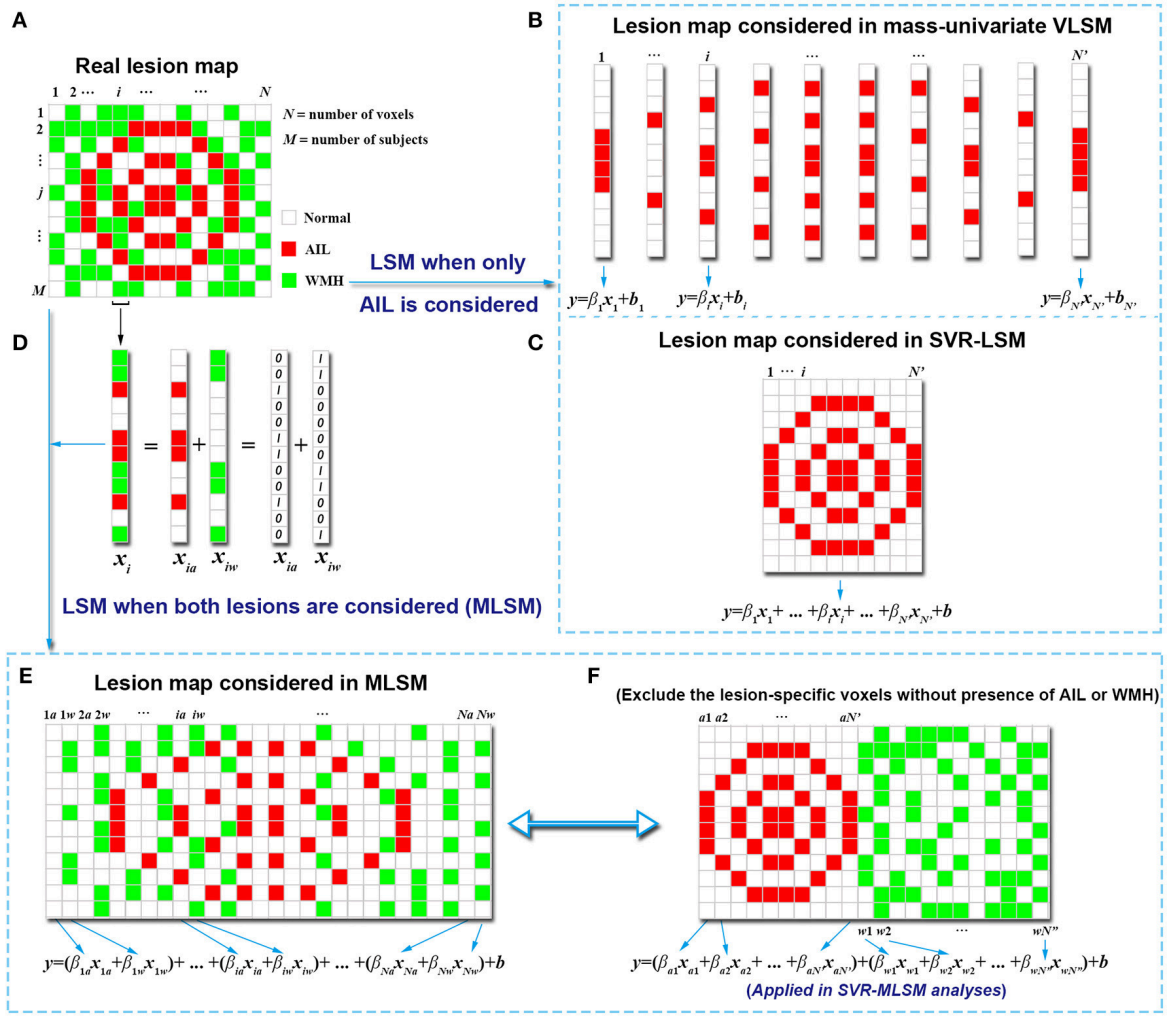
Simulation of model construction for MLSM. The simulated real lesion map **(A)** and the corresponding lesion maps considered in mass-univariate VLSM **(B)**, SVR-LSM **(C)** and MLSM **(E,F)** are provided. The way that MLSM considers the information of both lesions (AIL and WMH) is also illustrated **(D)**. The simulated lesion map **(A)** contains *N* voxels for each of the *M* subjects, with *N'* voxels lesioned by AIL in at least one subject, and with *N”* voxels lesioned by WMH in at least one subject (*N'*≤*N, N”*≤*N*).

Suppose that there are two kinds of lesions presented in a simulated image dataset as shown in Figure 1A. The real lesion map ***X*** contains *N* voxels and *M* subjects: *X* = (***x***_1_, ***x***_2_ ***x***_3_, …., ***x***_*N*_), where ***x***_*i*_ = (*x*_*i*1_, *x*_*i*2_, *x*_*i*3_ …, *x*_*iM*_) ^*T*^ indicates the lesion status of the *i*th voxel (*i* = 1, 2, …, *N*). Each voxel has three kinds of lesion status among the subjects: normal tissue, lesioned with AIL or lesioned with WMH.

In a mass-univariate VLSM model (the conventional VLSM) that only considers the presence of AIL, the lesion-symptom relationship for the *i*th voxel can be expressed as

(1) $$y=\beta_{i}x_{i}+b,$$

Where ***x***_*i*_ indicates the lesion status of the *i*th voxel for different subjects as damaged by AIL (with 1) or not (with 0), and ***y*** is the behavior score; β_*i*_ is the fitting coefficient and ***b*** are the fitting errors. Such a model is also constructed for the other voxels of the lesion map independently (Figure 1B). In contrast, a multivariate model, such as SVR-LSM (Zhang et al., 2014), considers the voxels as a whole lesion map and combines the lesion status of the voxels (damaged with AIL or not) in a single model (Figure 1C),

(2) $$y=\beta_{1}x_{1}+\beta_{2}x_{2}+\beta_{3}x_{3}+\cdots +\beta_{N}x_{N}+b.$$

As is shown in Figure 1, the information about the presence of WMH is not tested in both mass-univariate VLSM or SVR-LSM. The motivation for the MLSM model (multiple-lesion version of LSM) arises from the intention to account for more complete information of the lesion map for the LSM analyses where multiple kinds of lesions are presented. Here, the status of a voxel for a subject is exclusive in terms of lesion type, namely the voxel cannot be damaged with different lesions at the same time. To represent the lesion status in MLSM, we can identify each voxel in ***X*** with a single variable, which has different non-zero values that indicate different lesion status. However, it's difficult to allocate appropriate values for different lesion status and we can only do it empirically if possible (e.g., define 1 as lesioned with WMH, 2 as lesioned with AIL and 0 as normal for each voxel). More importantly, as the status of different lesions (nominal variable) would be treated as an ordinal variable in multiple regression, the impact of the locations of different lesions can hardly be separated in the analyses. Alternatively, each voxel in the lesion maps can be represented with separate variables in linear combinations, where each variable refers to the lesion status of one type of lesion for this voxel.

For example, if we consider both the presence of AIL and WMH in the multiple regression, each voxel ***x***_***i***_ in the real lesion map can be represented with two separate lesion-specific variables: ***x***_***ia***_ for AIL and ***x***_***iw***_ for WMH (Figure 1D). In detail, ***x***_***ia***_ indicates the status of the *i*th voxel with respect to AIL (damaged with AIL as 1 or not as 0), and ***x***_***iw***_ indicates the status of the *i*th voxel with respect to WMH (damaged with WMH as 1 or not as 0). In this way, we can model each voxel (e.g., the *i*th voxel for subject *m*) with three kinds of status: (1) *x*_*im,a*_ = 1 and *x*_*im,w*_ = 0 for the presence AIL, (2) *x*_*im, a*_ = 0 and *x*_*im,w*_ = 1 for the presence WMH, (3) *x*_*im,a*_ = 0 and *x*_*im,w*_ = 0 for the normal tissue. In addition, we can assign different weights to ***x***_***ia***_ and ***x***_***iw***_ to evaluate their impact on behavior separately in the multiple regression model. As such, Equation (2) can be adjusted to an MLSM model as follows:

(3) $$\begin{array}{l} y=(\beta_{1a}x_{1a}+\beta_{1w}x_{1w})+(\beta_{2a}x_{2a}+\beta_{2w}x_{2w})+\cdots \\ \;+(\beta_{Na}x_{Na}+\beta_{Nw}x_{Nw})+b. \end{array}$$

This model corresponds to the lesion map shown in Figure 1E. As not all voxelwise variables (***x***_***i***_) involve both the presence of AIL and WMH among the subjects (for example when ***x***_***ia***_ or ***x***_***iw***_ is a zero vector), we can exclude the lesion-specific variables that carry no information (blank columns in Figure 1E) and simplify the model as follows (Figure 1F),

(4) $$\begin{array}{l} y=(\beta_{a1}x_{a1}+\beta_{a2}x_{a2}+\cdots +\beta_{aN^{\prime }}x_{aN^{\prime }})\\ \;+(\beta_{w1}x_{w1}+\beta_{w2}x_{w2}+\cdots +\beta_{wN^{\prime \prime }}x_{wN^{\prime \prime }})+b. \end{array}$$

Here, *N'* indicates the number of voxels damaged with AIL in at least one subject and *N”* indicates the number of voxels damaged with WMH in at least one subject (*N'*≤*N, N”*≤*N*). The number of the voxels for AIL and WMH considered in the real analysis should be even smaller, as the general criteria that a voxel can be included in the LSM analysis is that it's damaged in at least three to five or even more subjects (Biesbroek et al., 2015).

Equation (4) can also be simplified as

(5) $$y=\beta_{a}X_{a}+\beta_{w}X_{w}+b,$$

Where ***X***_***a***_ = (***x***_***a***1_, ***x***_***a***2_, …, ***x***_***aN***′_)^*T*^ and **β**_**a**_ = (β_*a*1_, β_*a*2_, …, β_*aN*_′) indicate the lesion map and corresponding weighting coefficients with respect to AIL, and ***X***_***w***_ = (***x***_***w***1_, ***x***_***w***2_, …, ***x***_***wN***″_)^*T*^ and **β**_***w***_ = (β_*w*1_, β_*w*2_, …, β_*wN*″_) with respect to WMH. As such, the lesion maps of AIL and WMH are combined as an entire lesion map in the MLSM model (Figure 1F) that represents the complete information of the real lesion map (Figure 1A). To calculate the beta coefficients and their significance levels, we applied support vector regression as detailed in the next section.

### MLSM through support vector regression (SVR-MLSM)

Solving the multiple regression model as Equation (5) is difficult due to the collinearity of the adjacent voxels and the under-determinacy caused by the much greater number of voxels (unknown variables) than the number of subjects (observations) (Zhang et al., 2014). Furthermore, for a voxel ***x***_***i***_, its lesion-specific variable ***x***_***ia***_ (for AIL) and ***x***_***iw***_ (for WMH) might also be highly correlated (as the presence of AIL and the presence of WMH on a voxel is exclusive for a certain subject), which further improves the multicollinearity in the model. However, the machine learning approaches that are not sensitive to multicollinearity, such as support vector regression (SVR), will be able to highlight the most significant lesion status for each of the voxels. Therefore, we adjusted the recently developed SVR-LSM approach (Zhang et al., 2014) to a multiple-lesion version (named SVR-MLSM) with the intuition in Equation (5) to investigate the association between the locations of AIL and WMH and post-stroke cognition.

The published SVR-LSM model for a single lesion type (Zhang et al., 2014) was nonlinear, which helped to map the lesion-behavior correlations to a high dimensional space. To project this high dimensional relationship back to a linear space for the statistical inference, the authors performed volume control by weighting the lesioned voxels (***X***) in inverse proportion to the square root of the lesion size for each subject, and they assumed that the total lesion burden is large enough to make the normalized-value of each voxel (*x*_*i, j*_ and *x*_*j*_ as in Equation 6) in the lesion maps very close to zero. In this regard, the nonlinear model of Equation (6) in the SVR-LSM paper can be expressed as a linear form with the approximation based on the first order Taylor expansion (Zhang et al., 2014).

(6) $$y=\sum_{j}\sum_{i}\lambda_{i}\cdot e^{-\gamma {\left\|x_{i,j}-x_{j}\right\|}^{2}}$$

When it comes to the MLSM problem, this approximation cannot be guaranteed. When different kinds of lesions are considered in the same model, the volume control cannot be simply performed by weighting the voxels with the sum of the total AIL volume and the total WMH volume. Even if the lesion-specific variables ***X***_***a***_ and ***X***_***w***_ were normalized by the lesion size of AIL and WMH respectively, the equivalent multiple-lesion version of Equation (6) still cannot be approximated to a linear form, because the normalized values for each voxel in ***X***_***a***_ and ***X***_***w***_ can hardly be balanced to achieve a similar first order Taylor expansion-based approximation as in nonlinear SVR-LSM. Therefore, we adjusted the nonlinear SVR model to a linear model for the SVR-MLSM analysis considering the feasibility for statistical inference.

Without volume control, LSM might unintentionally identify brain regions that are related to larger lesion size, but not to the symptom of interest itself (Sperber and Karnath, 2017). In this case, volume control is generally performed in both mass-univariate VLSM and SVR-LSM studies. Regarding volume control for the SVR-MLSM, it can be achieved by normalizing the variables in ***X***_***a***_ by the lesion size of AIL and those in ***X***_***w***_ by the lesion size of WMH for each subject. In addition, volume control by regressing out the AIL volume and WMH volume should also be attempted as it enables the direct comparison between the voxelwise weight coefficients of different lesion types (**β**_***a***_ and **β**_***w***_), which is not available for the voxelwise normalization-based volume control.

The statistical inference of SVR-MLSM was similar with that of SVR-LSM, which was realized by shuffling the observations of the behavior scores to create pseudo weight coefficients, and the significance level of each voxel was calculated by counting the number of pseudo weights larger than the real weight in the permutations (if the behavior score is positively correlated with the deficits). The only difference is that in SVR-MLSM the permutation-based pseudo weights of AIL and WMH voxels are generated at the same time, and we can identify the strategic voxels of AIL and WMH with the same significance level by permutations. To be consistent with the SVR-LSM methodology paper (Zhang et al., 2014), where the behavior score is an index of deficits, we will perform a linear transformation for the behavior score prior to the model training when the behavior score (e.g., MoCA) is negatively correlated with the deficits (Zhao et al., 2017).

## Materials and methods

### Subjects

Participants were patients of the ongoing Chinese University - Stroke Registry Investigating Cognitive Decline (CU-STRIDE) study (Yang et al., 2015). The CU-STRIDE study recruited 1,013 consecutive acute stroke/TIA patients, who were admitted to the Prince of Wales Hospital in Hong Kong between 2009 and 2010, aiming to investigate mechanisms of cognitive decline over 5 years. The CU-STRIDE study obtained the approval from the Joint Chinese University of Hong Kong – New Territories East Cluster Clinical Research Ethics Committee with written informed consent from all participants. The inclusion and exclusion criterion of the CU-STRIDE trial were described previously (Yang et al., 2015). Among the 510 patients who were examined by MRI, we further excluded the patients without visible AILs on diffusion-weighted imaging (DWI) and those not available for fluid-attenuated inversion recovery (FLAIR) scans.

As we intended to associate both locations of AILs and WMHs and post-stroke cognition in this study, which was a relatively complex issue, we need to purify the study sample for subsequent analyses and interpretations. Firstly, we expect that the presence of old infarcts at baseline would be a strong confounder when we investigate the association between WMH locations and post-stroke outcomes. Therefore, the patients with prior stroke were excluded. Secondly, although both AIL and WMH locations were considered, the rough AIL locations (infratentorial or supratentorial, right or left) should be purified for easier interpretations of the MLSM results. In this regard, we further excluded the patients with infratentorial AIL [less important than supratentorial stroke for cognitive impairment (Bastos Leite et al., 2006)] and those with right supratentorial AIL [generally with better recovery in cognition than patients with AIL in the left cerebral (Hochstenbach et al., 2003)].

**Figure 2:**
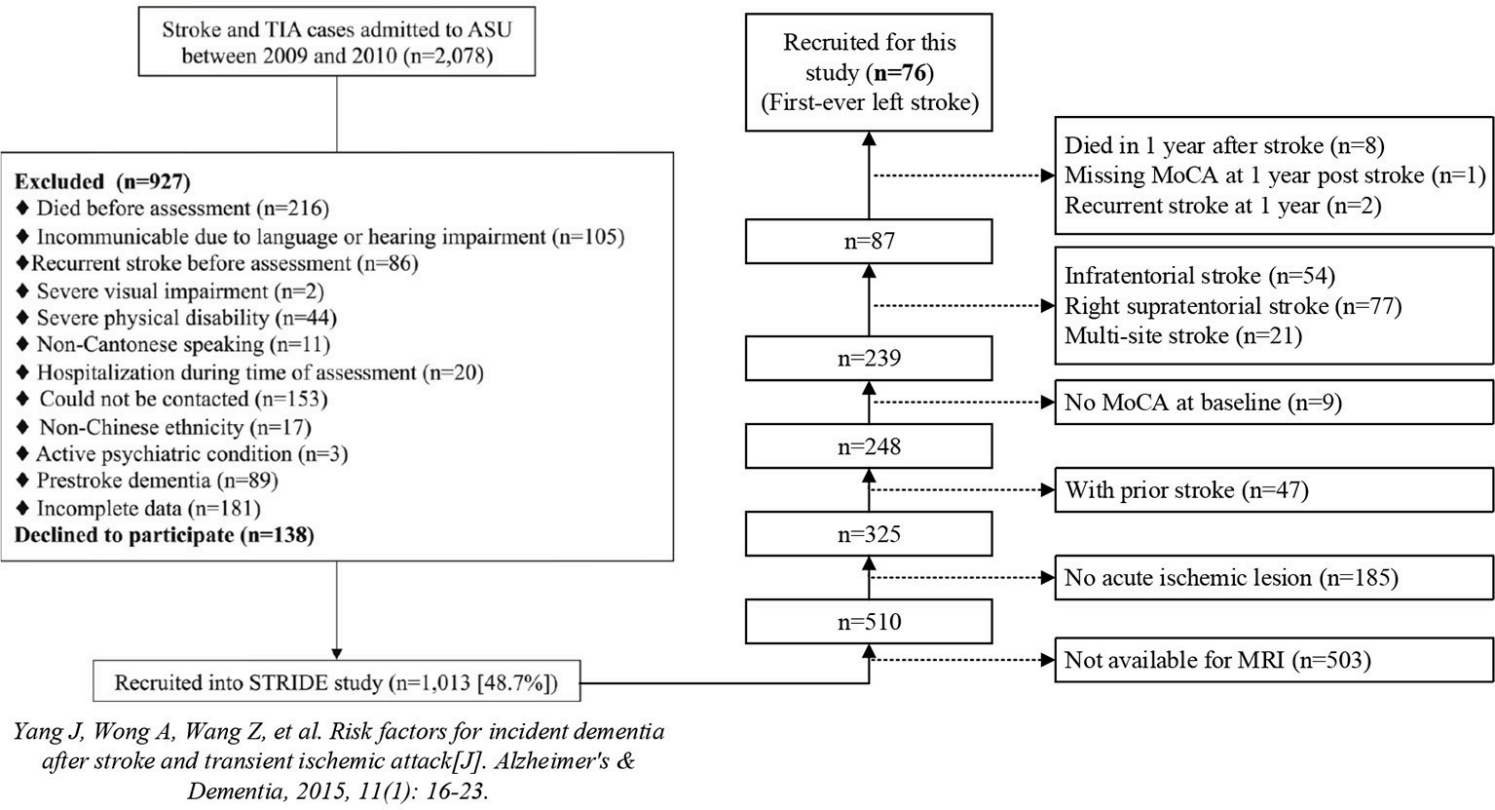
Flowchart of patient inclusion.

In addition, the patients who were not available for cognitive assessment of the Hong Kong version of Montreal Cognitive Assessment (MoCA) (Wong et al., 2009) both at baseline (3–6 months after the first onset of stroke as short-term outcome) and 1 year (15–18 months after the first onset of stroke as long-term outcome) post stroke were also excluded. Furthermore, we excluded the patients with recurrent stroke at 1 year after baseline stroke to make the long-term lesion-symptom mapping more reasonable, because there would be great changes to the brains with recurrent stroke compared to the baseline images. Finally, the remaining 76 patients with first-ever stroke in the left cerebral were included in this study (Figure 2).

### Generation of lesion maps

Brain MRI examinations were performed for the patients within 1 week of hospital admission on a 1.5T scanner (Sonata; Siemens Medical, Erlangen, Germany) or a 3.0T scanner (Achieva 3.0T TX Series; Philips Medical System, Best, the Netherlands) using standard protocols (Yang et al., 2015). The applied MRI sequences in the proposed study included DWI, axial FLAIR and axial spin echo T1-weighted fast field echo, and their imaging parameters were previously described (Yang et al., 2015). AILs were manually delineated on DWI, and WMHs were automatically segmented using a previously published approach (Shi et al., 2013) with manual correction when needed. The DWI and FLAIR sequences were first linearly registered to the T1 sequences of the patients and were further registered to the 1-mm T1 MNI-152 (Montreal Neurological Institute) template (Fonov et al., 2009). The registration procedure was performed with a linear registration followed by a non-linear registration using elastix (Klein et al., 2010), and the resulting transformations were combined to transform the corresponding lesion maps of AIL and WMHs to the MNI-152 template. Rigorous quality checks of the registration results were performed by comparing the location of the lesion maps of AIL and WMH on native scan to that on MNI-152 template. Manual correction of the mapped lesions was performed when necessary. The lesion size of AIL and WMH were both calculated in the MNI space and used in the subsequent analyses.

### LSM analyses with mass-univariate VLSM, SVR-LSM and SVR-MLSM

The mass-univariate VLSM was performed to associate the presence of AIL in each voxel and the norm-corrected MoCA scores. Voxels affected by AIL in less than three patients were not considered for the analysis, and this threshold has been used in previous studies with a sample size of less than one hundred subjects (Buiatti et al., 2012; Tsuchida and Fellows, 2013; Biesbroek et al., 2015, 2016). The baseline and year 1 MoCA scores were norm-corrected for age, gender and education year. Parametric *t*-test with multiple comparison corrections based on 1,000 permutations was used for statistical inference. Here, we selected permutation test rather than FDR or Bonferroni correction to make the statistical inference procedure consistent with that of SVR-LSM and SVR-MLSM analyses. Considering the volume control issue, we prepared two models for mass-univariate VLSM. The total volume of AIL was additionally regressed out from the MoCA scores for the mass-univariate VLSM model with volume control. As regressing out the lesion size may reduce the statistical power (Karnath et al., 2004), we also tested the model without volume control. For these two mass-univariate VLSM models, the voxels with *p* < 0.05 after the 1,000 permutations were treated as significant.

Regarding the SVR-LSM and SVR-MLSM analyses, we designed several models with different volume control strategies to have a more comprehensive comparison of these methods. In the published SVR-LSM paper (Zhang et al., 2014), the SVR-LSM model was nonlinear with voxelwise normalization as the volume control. To make an equivalent comparison with the SVR-MLSM where only linear kernel can be used, we additionally performed linear SVR-LSM with the similar volume control strategies for SVR-MLSM. Finally, there were four SVR-LSM models for comparison: (1) nonlinear model with voxelwise volume control (Zhang et al., 2014); (2) linear model without volume control; (3) linear model with voxelwise volume control by weighting the voxels in inverse proportion to the square root of the AIL volume (Zhao et al., 2017); (4) linear model with volume control by regressing out the AIL volume from MoCA. In line with the mass-univariate VLSM models, we only considered the voxels damaged with AIL in at least three patients in the SVR-LSM analyses. The MoCA scores were norm-corrected for age, gender and education year [and lesion size of AIL only for volume control strategy (4)] and then transformed to deficit scores.

In the SVR-MLSM (with linear kernel) analyses, we selected three models as follows: (1) without volume control; (2) volume control with voxelwise normalization, namely by weighting the voxels of AIL (***X***_***a***_) in inverse proportion to the square root of the AIL volume and the voxels of WMH (***X***_***w***_) in inverse proportion to the square root of the WMH volume; (3) volume control by regressing out the AIL volume and WMH volume from MoCA. In the SVR-MLSM analyses, we also only considered the voxels damaged with AIL in at least three patients for ***X***_***a***_ and the voxels damaged with WMH in at least three patients for ***X***_***w***_. We norm-corrected the MoCA scores at baseline and 1 year after stroke for age, gender and education year [and the lesion size of AIL and WMH only for volume control strategy (3)] and then transformed the norm-corrected scores to deficit scores.

The analyses with SVR-LSM and SVR-MLSM were realized by model training and subsequent statistical inference. The model training was applied to optimize the prediction accuracy of an SVR model by searching the model parameters during cross-validations. Here, the lesion map ***X*** preprocessed by different volume control strategies and the behavior score ***y*** preprocessed by norm-correction were entered in the SVR-LSM or SVR-MLSM models, and parameter optimization with leave-one-out cross-validation was performed for these SVR models. In detail, using a specific set of parameter(s) and within the 76 observations (subjects), each time we selected one observation for testing and the remaining 75 for training until all the observations had been used in the testings. In this way, 76 predicted behavior scores were generated, and the prediction accuracy of an SVR-LSM or SVR-MLSM model with this set of parameter(s) was calculated as the Pearson correlation coefficient between the predicted behavior scores and the real behavior scores (Yourganov et al., 2016). During the parameter training, we searched C (box constraint) from 2^−20^ to 2^20^ that achieved best prediction accuracy for the corresponding linear SVR-MLSM and linear SVR-LSM models, and searched C from 2^−20^ to 2^6^ and γ (kernel scale) from 1 to 16 for the nonlinear SVR-LSM with radial basis function (RBF) kernel. Here, the box constraint (C) controls the trade-off between the flatness and the tolerable fitting error, and the kernel scale (γ, the free parameter of the Gaussian radial basis function in a nonlinear model) controls the trade-off between error due to bias and variance. With the optimized parameter(s) for a specific SVR model, we generated the real weight coefficients for the voxels in the lesion map. Then we performed statistical inference by shuffling the observations of MoCA deficit scores to create pseudo weight coefficients, and the significance level of each voxel was calculated by counting the number of pseudo weights larger than the real weight in 1,000 permutations. In line with the mass-univariate VLSM models, significant clusters generated from SVR-LSM and SVR-MLSM should survive the threshold of *p* < 0.05 in the 1,000 permutations.

### Behavior prediction based on the results of the LSM methods

The performance of the SVR-MLSM and SVR-LSM models can be compared by the optimal prediction accuracy derived from leave-one-out cross-validations during parameter training. However, this kind of comparison is not available for the mass-univariate VLSM due to the difference in their statistical inference procedures. As all these LSM methods shared the objective to highlight the significant anatomical regions for a specific behavior, we can alternatively compare their behavior prediction performance based on the significant clusters from these models that are generated by statistical inference. Specifically, we can combine the significant clusters from a specific LSM model as a single region and calculate the lesion volume of the subjects that overlap with this significant anatomical region (Forkert et al., 2015; Munsch et al., 2016). In the following context, we defined this measure as the significant clusters-based volume of interest (SVOI).

To compare the behavior prediction power of the SVOI from different LSM methods, we used the SVOI of AIL (SVOI-AIL) and/or WMH (SVOI-WMH) and some covariates as independent variables and the raw MoCA score as the dependent variable in a linear SVR model. In detail, in the SVR model with SVOI generated from mass-univariate VLSM or SVR-LSM, demographic variables (age, gender, education year), lesion size of AIL and SVOI-AIL were entered as independent variables. And in the SVR model with SVOI generated from SVR-MLSM, demographic variables, lesion size of AIL, lesion size of WMH, SVOI-AIL and SVOI-WMH were entered as independent variables. Several models with only demographic variables and lesion size were also attempted for comparison. The model training procedure here was similar with that of SVR-LSM or SVR-MLSM. The prediction accuracy of each SVR model was measured by the Pearson correlation coefficient between the predicted behavior scores and the real behavior scores, and it was optimized through parameter training during leave-one-out cross-validation. The contribution of SVOI-AIL and SVOI-WMH can be measured by the change in prediction accuracy of MoCA scores before and after adding them as independent variables in the SVR models. The SVR-MLSM model that gained best prediction accuracy of both baseline and year 1 MoCA scores (with its SVOI-AIL and SVOI-WMH) will be selected for the subsequent comparison of the significant lesion locations for short-term and long-term outcomes.

## Results

### Patient characteristics

**Figure 3:**
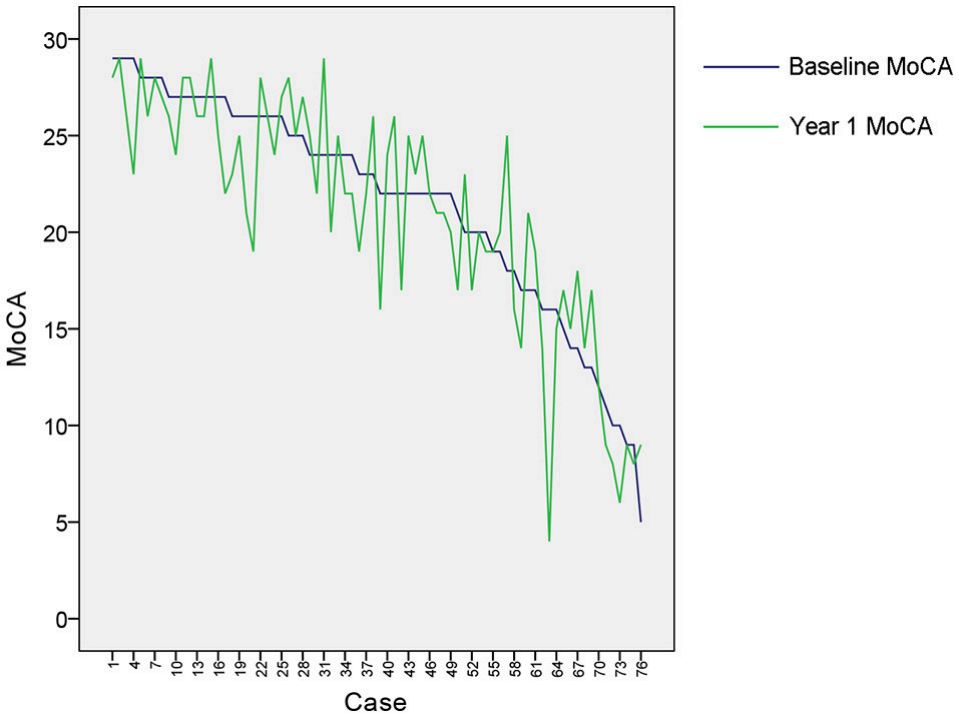
Cognitive changes for the patients. The baseline MoCA scores were sorted in descending order for the included cases, and year 1 MoCA scores were subsequently displayed for these cases correspondingly.

**Figure 4:**
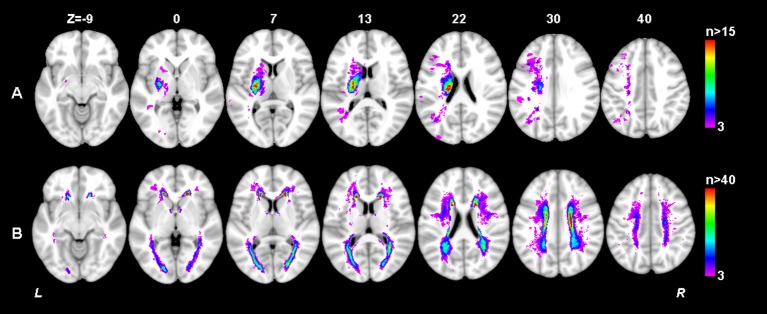
Lesion prevalence of acute ischemic lesion **(A)** and white matter hyperintensity **(B)**. Voxels that are damaged in at least three patients are projected on the 1mm MNI-152 template (Z coordinates: −9, 0, 7, 13, 22, 30, 40). Bar indicates the number of patients with a lesion for each voxel.

**Figure 5:**
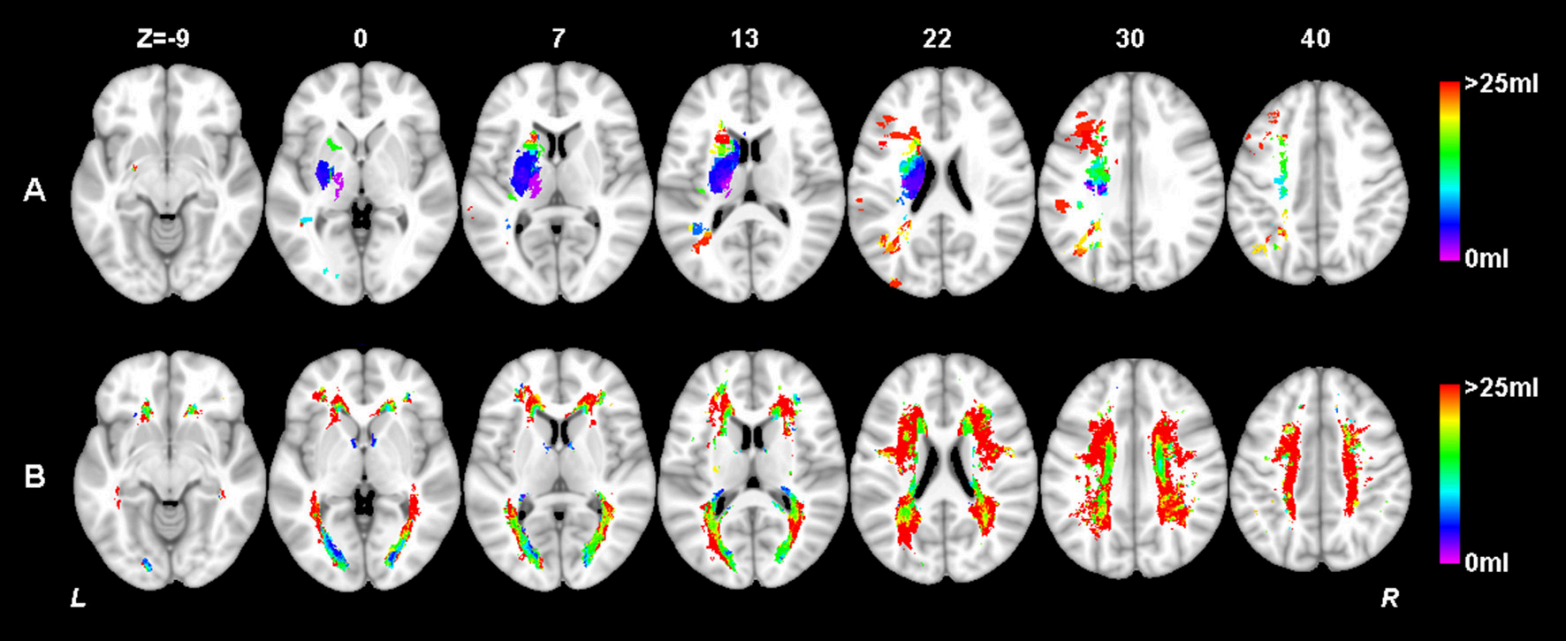
Lesion size topologies of acute ischemic lesion **(A)** and white matter hyperintensity **(B)**. Bar indicates the median acute infarct volume **(A)** or median white matter hyperintensity volume **(B)** a patient would have, given that the specific voxel is lesioned.

Clinical characteristics of the patients in this study are provided in Table 1. Although the mean education level (6.8 years) was relatively low and might limit the ability to estimate cognition using standardized testing, we have validated the Hong Kong version of the MoCA against a group of patients with a mean education of 5.9 years and it has been shown that this test is a reliable and useful cognitive screening instrument in patients with small vessel disease (Wong et al., 2009). Although the distribution of MoCA scores at baseline and 1 year after stroke appeared similar as shown in Table 1, most of the patients had cognitive changes (either decline or recovery) at follow-up as shown in Figure 3. In fact, more than half (51.3%) of the patients had cognitive decline in global cognition at 1 year post stroke compared to their baseline performance, which would help to investigate the varied significant regions for short-term and long-term outcomes. The median AIL volume was 2.10 ml, indicating that the majority of patients had relatively small acute infarcts rather than large infarcts. The distribution of AIL and WMH in the study cohort were illustrated by the lesion prevalence maps in Figure 4. Lesion prevalence of AIL (Figure 4A) was higher in the basal ganglia than the white matter and cortex, and the prevalence of periventricular WMH (Figure 4B) was higher than deep WMH. In addition, Figure 5 illustrated the lesion size topologies (Sperber and Karnath, 2016) of AIL and WMH in the study cohort. Although the lesion distribution of WMH appeared to be similar with that in normal aging subjects, we demonstrated in the Supplementary Materials (Figures S1, S2 and Table S1) that the lesion size of WMH was larger and the distribution of WMH spread more to the deep white matter in our stroke cohort than a normal aging cohort (well-matched in age, gender and education level).

**Table 1:** Characteristics of the study cohort.

**Characteristics**	**Study cohort (*n* = 76)**
**DEMOGRAPHIC CHARACTERISTICS**	
Age, mean ± SD (years)	65.8 ± 10.1
Education, mean ± SD (years)	6.8 ± 4.2
Female, n (%)	32 (42.1)
**HANDEDNESS**	
Right, n (%)	75 (98.7)
Ambidextrous, n (%)	1 (1.3)
**STROKE SUBTYPE**	
Large-artery atherosclerosis, n (%)	47 (61.8)
Small-artery occlusion, n (%)	21 (27.6)
Cardioembolism, n (%)	6 (7.9)
Others, n (%)	2 (2.6)
**VASCULAR RISK FACTORS**	
Smoking, n (%)	17 (22.4)
Hypertension, n (%)	55 (72.4)
Diabetes mellitus, n (%)	27 (35.5)
**LESION MEASURES**	
Median acute infarct volume, ml (range)	2.10 (0.14-62.99)
Median white matter hyperintensity volume, ml (range)	8.59 (1.47-55.89)
**COGNITIVE MEASURES**	
Baseline MoCA, mean ± SD	21.6 ± 5.7
Year 1 MoCA, mean ± SD	21.1 ± 6.1
Year 1 MoCA < baseline MoCA, n (%)	39 (51.3)

### Parameter training of SVR-LSM and SVR-MLSM models

The optimized parameters and prediction performance of the SVR-LSM and SVR-MLSM models were shown in Table 2. The prediction accuracy based on leave-one-out cross-validation during parameter training was generally higher in SVR-LSM models than that in SVR-MLSM models. When volume control was not performed, or performed by weighting each voxel in the lesion map, the linear SVR-LSM model with no volume control best predicted the norm-corrected baseline MoCA, and the SVR-MLSM model with no volume control best predicted the norm-corrected year 1 MoCA. When the lesion size was additionally regressed out from the norm-corrected MoCA scores, the corresponding SVR-LSM and SVR-MLSM models were inferior to the other models in prediction accuracy.

**Table 2:** Optimized parameters and prediction accuracy of SVR-LSM and SVR-MLSM models.

	**Model**	**Baseline MoCA**			**Year 1 MoCA**		
		**Prediction accuracy**	***p***	**Parameters**	**Prediction accuracy**	***p***	**Parameters**
SVR-LSM with AIL	Linear - no volume control	0.4062	<0.001	c = 2^−13^	0.3788	<0.001	c = 2^−10^
	Linear - voxelwise normalization	0.2813	0.014	c = 4	0.3418	0.003	c = 2
	Linear - total volume regressed out^*^	0.2753	0.016	c = 2^−11^	0.1642	0.156	c = 2^−3^
	Nonlinear - voxelwise normalization	0.3715	<0.001	γ = 8, c = 2^−20^	0.3648	0.001	γ = 8, c = 2^−20^
MLSM with AIL and WMH	Linear - no volume control	0.3097	0.007	c = 2^−17^	0.4033	<0.001	c = 2^−12^
	Linear - voxelwise normalization	0.2509	0.029	c = 0.25	0.2899	0.011	c = 0.5
	Linear - total volumes regressed out^∧^	0.2538	0.027	c = 2^−16^	0.1818	0.116	c = 2^−16^

* *The total lesion burden of AIL was regressed out from the baseline and year 1 MoCA in this SVR-LSM model*.

∧ *The total volume of AIL and that of WMH were regressed out from the baseline and year 1 MoCA in this SVR-MLSM model*.

### Behavior prediction based on significant lesion locations from LSM analyses

The results of behavior prediction based on the significant AIL locations (for mass-univariate VLSM, SVR-LSM, and SVR-MLSM) and WMH locations (for SVR-MLSM) were shown in Table 3. The visualization of the significant clusters generated by different LSM models were also provided in Figures S3, S4. The total lesion size of AIL (Model 2) had little contribution to the prediction of baseline MoCA and it improved the prediction accuracy of year 1 MoCA by 9.24% compared to the model with only demographic variables (Model 1). The lesion size of WMH (Model 3) contributed little to both short-term and long-term outcomes on top of the lesion size of AIL.

**Table 3:** Behavior prediction based on the significant clusters of mass-univariate VLSM, SVR-LSM, and SVR-MLSM.

**Model**	**Independent variables**	**Baseline MoCA**		**Year 1 MoCA**	
		**Accuracy**	***p***	**Accuracy**	***p***
1	Age, gender, education year	0.5897	2.08E-08	0.6075	5.94E-09
2	Model 1 + Total infarct volume	0.6219	2.02E-09	0.6999	1.99E-12
3	Model 2 + Total WMH volume	0.6020	8.83E-09	0.7108	6.33E-13
4	Model 2 + VLSM SVOI-AIL (noVol)	0.7173	3.10E-13	0.7208	2.10E-13
5	Model 2 + VLSM SVOI-AIL (totalVol)	0.6214	2.11E-09	0.7000	1.98E-12
6	Model 2 + SVR-LSM SVOI-AIL (noVol)	0.7328	5.31E-14	0.7067	9.82E-13
7	Model 2 + SVR-LSM SVOI-AIL (voxelwise)	0.7264	1.12E-13	0.7428	1.57E-14
8	Model 2 + SVR-LSM SVOI-AIL (totalVol)	0.6779	1.73E-11	0.7121	5.49E-13
9	Model 2 + SVR-LSM SVOI-AIL (nonlinear)	0.7009	1.80E-12	0.7355	3.81E-14
10	Model 3 + MLSM SVOI-AIL (noVol)	0.7026	1.50E-12	0.7290	8.25E-14
11	Model 3 + MLSM SVOI-AIL (voxelwise)	0.7074	9.07E-13	0.7593	1.89E-15
12	Model 3 + MLSM SVOI-AIL (totalVol)	0.7086	8.01E-13	0.7622	1.27E-15
13	Model 3 + MLSM SVOI-WMH (noVol)	0.7935	1.26E-17	0.8140	3.91E-19
14	Model 3 + MLSM SVOI-WMH (voxelwise)	0.8467	5.71E-22	0.8525	1.52E-22
15	Model 3 + MLSM SVOI-WMH (totalVol)	0.7906	2.00E-17	0.8730	8.89E-25
16	Model 3 + MLSM SVOI-AIL + MLSM SVOI-WMH (noVol)	0.8237	6.58E-20	0.8443	9.70E-22
17	Model 3 + MLSM SVOI-AIL + MLSM SVOI-WMH (voxelwise)	0.8600	2.56E-23	0.8826	5.74E-26
18	Model 3 + MLSM SVOI-AIL + MLSM SVOI-WMH (totalVol)	0.8112	6.42E-19	0.8750	5.10E-25

*The prediction performance was evaluated using support vector regression through leave-one-out cross-validation. The prediction accuracy was calculated as the Pearson correlation coefficient of the real MoCA score and the predicted MoCA score, and the corresponding p-value was also provided. SVOI, significant clusters-based volume of interest; noVol, without volume control; voxelwise, voxelwise normalization by weighting each voxel with inverse proportion to the square root of the corresponding lesion size; totalVol, volume control by regressing out the total lesion size from baseline or year 1 MoCA*.

Regarding the variables of SVOI-AIL generated by mass-univariate VLSM and SVR-LSM (Model 4 ~ Model 9), they generally had significant contribution to baseline MoCA and improved the prediction accuracy of baseline MoCA by about 10% on top of the AIL size and demographic variables (Model 2), but had little contribution to year 1 MoCA, where the most improvement (4.29%) was achieved by the SVOI-AIL from the SVR-LSM model with voxelwise volume control (Model 7). In addition, the linear SVR-LSM with voxelwise volume control (Model 7, with the best performance among the SVR-LSM models) performed a little better than the mass-univariate VLSM models (Model 4 and Model 5) in both predictions of short-term and long-term outcomes.

The SVOI-AIL generated by SVR-MLSM models (Model 10 ~ Model 12) performed similar with those from the SVR-LSM models (Model 6 ~ Model 9). When the SVOI-WMH generated from SVR-MLSM was considered alone, the corresponding models (Model 13 ~ Model 15) generally improved the prediction accuracy of both short-term and long-term outcomes by over 10% compared with the models with only SVOI-AIL considered (Model 4 ~ Model 12). With both SVOI-AIL and SVOI-WMH from SVR-MLSM considered, the prediction accuracy was further slightly improved as shown in Model 16 ~ Model 18. Specifically, the SVR-MLSM model with voxelwise volume control (Model 17) achieved the best performance in the behavior prediction with its SVOI-AIL and SVOI-WMH (86.00% for baseline MoCA and 88.26% for year 1 MoCA), and it was used to compare the significant AIL and WMH locations for short-term and long-term outcomes in the subsequent analyses.

### SVR-MLSM results for baseline and follow-up outcomes

**Figure 6:**
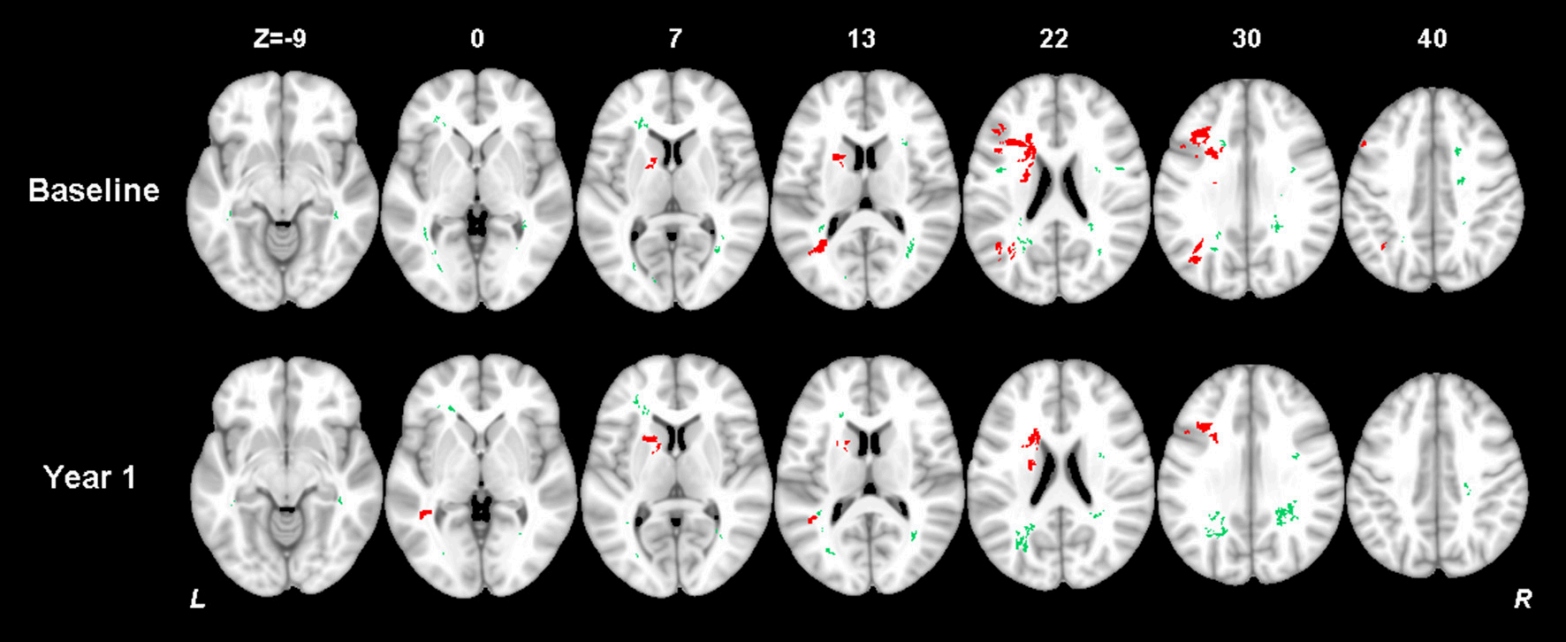
Results of multiple-lesion symptom mapping (the SVR-MLSM model with volume control by voxelwise normalization, Model 17 in Table 3). Voxelwise associations between the presence of a lesion (AIL or WMH) and global cognition at baseline and 1 year after stroke were determined using SVR-MLSM. This multivariate approach assesses inter-voxel and inter-lesion correlations and identifies the voxels of AIL or WMH which have an independent contribution to the outcome. The significant clusters of AIL (in red) and WMH (in green) were shown with *p* < 0.05 from statistical inference based on 1,000 permutations.

The thresholded statistical results of the optimal SVR-MLSM model (with voxelwise volume control, Model 17 in Table 3) were shown in Figure 6. The clusters of the AIL (in red) and WMH (in green) were thresholded with *p* < 0.05 from the statistical inference based on 1,000 permutations. The corresponding beta (voxelwise fitting coefficient) maps for the cognitive impairment at baseline and 1 year after stroke were shown in Figures S5, S6. The significant clusters of AIL were mapped to the AAL and ICBM-DTI-81 atlases in Table S2, and the significant clusters of WMH were mapped to ICBM-DTI-81 atlas in Table 4.

**Table 4:** SVR-MLSM results of white matter hyperintensities.

**Region**	**Patients with lesion (n)^∧^**	**Region size in voxel (n)**	**Tested voxels (n)**	**Significant voxels of WMH [n (%)]**	
				**Baseline MoCA**	**Year 1 MoCA**
Body of corpus callosum	45	17,849	4,621	83 (1.80)	0
Splenium of corpus callosum^*^	36	19,535	4,610	329 (7.14)	363 (7.87)
Anterior corona radiata L^*^	29	7,507	3,428	154 (4.49)	192 (5.60)
Superior corona radiata L	40	8,929	6,886	34 (0.49)	0
Superior corona radiata R	52	8,759	6,062	219 (3.61)	40 (0.66)
Posterior corona radiata L^*^	35	5,325	3,766	157 (4.17)	324 (8.60)
Posterior corona radiata R^*^	49	5,953	4,670	152 (3.25)	383 (8.20)
Posterior thalamic radiation L^*^	27	6,387	3,391	149 (4.39)	347 (10.23)
Posterior thalamic radiation R	32	5,400	3,335	398 (11.93)	142 (4.26)
Sagittal stratum L^*^	12	2,184	280	76 (27.14)	99 (35.36)
Sagittal stratum R	14	2,173	415	164 (39.52)	99 (23.86)
Superior longitudinal fasciculus L	14	9,386	2,045	64 (3.13)	0
Superior longitudinal fasciculus R^*^	11	9,580	2,737	0	55 (2.01)
Tapetum R	36	663	613	36 (5.87)	0

Regions where there were significant WMH clusters (p < 0.05) for global cognition at baseline and 1 year after stroke. The remaining regions in ICBM-DTI-81 white matter tract atlas contained no significant voxels either for baseline or 1 year cognitive impairment; these regions are not shown here. L, left; R, right.

∧ *Number among 76 included patients had WMH that overlapped (≥1 voxel) with the specified region of interest in ICBM-DTI-81 atlas*.

* *Regions where more WMH clusters were significantly associated with the long-term cognitive impairment than short-term cognitive impairment*.

**Figure 7:**
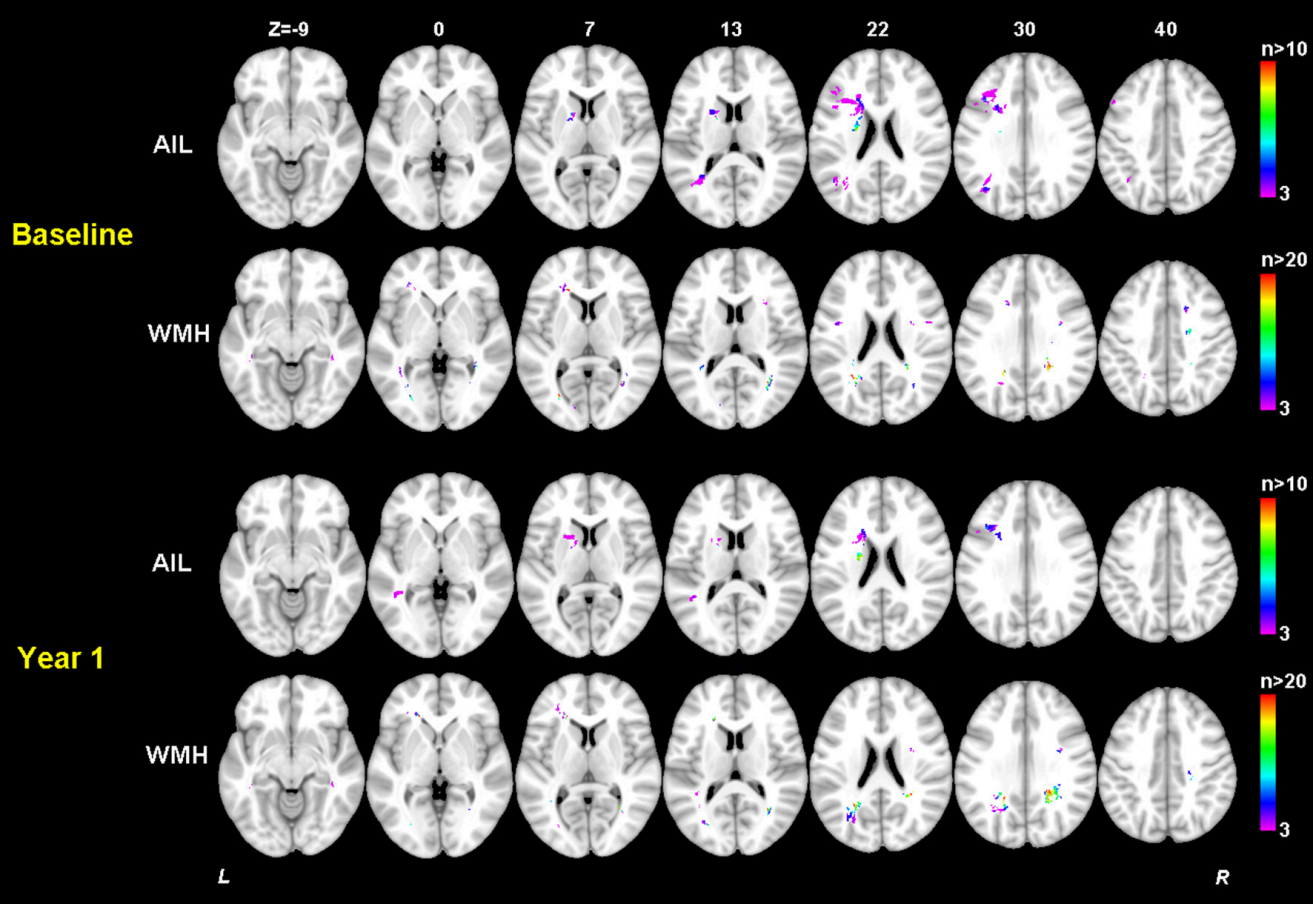
Number of patients with a lesion in each of the significant voxels of AIL or WMH from the SVR-MLSM analyses (corresponding to Model 17 in Table 3). Bar indicates the number of patients with a lesion for each voxel. The figures are shown in neurological convention (left is on the left).

The significant AIL locations for the baseline cognitive dysfunction were mainly in the left basal ganglia, left frontal, temporal and occipital cortex and white matter. At 1 year after stroke, the significant AIL locations appeared as a subset of those significant AIL clusters at baseline (Figure 6 and Table S2), and most associated with the cognitive dysfunction were the AILs in the left basal ganglia. The significant WMH locations were generally similar at baseline and at 1 year after stroke, which were mainly in the corpus callosum, corona radiata, and posterior thalamic radiation, and there were larger contralesional clusters in the posterior white matter at 1 year after stroke compared with baseline (Table 4). The significant clusters of AIL and WMH were also interleaved with the lesion prevalence maps to show the number of patients the significant results were based on (Figure 7). The results of AIL were more biased to the voxels with only three to five patients involved than the results of WMH. By comparing the significant regions shared by AIL and WMH (Table 5), we found that in some regions, the AIL clusters that were significant for short-term outcome were no longer significant for long-term outcome, and interestingly more WMH clusters in these regions became significant for the long-term outcome compared to short-term outcome. For example, as was shown in slice *Z* = 22 and *Z* = 30, the significant AIL clusters at baseline in the left parietal white matter were no longer significant at 1 year after stroke, while the size of significant WMH clusters increased in this region for the long-term outcome compared to baseline (Figure 6).

**Table 5:** Significant regions shared by AIL and WMH from SVR-MLSM analyses.

**Region**	**Significant voxels of AIL [n (%)]**		**Significant voxels of WMH [n (%)]**	
	**Baseline MoCA**	**Year 1 MoCA**	**Baseline MoCA**	**Year 1 MoCA**
Body of corpus callosum	60 (13.36)	36 (8.02)	83 (1.80)	0
Anterior corona radiata L^*^	413 (55.28)	0	154 (4.49)	192 (5.60)
Superior corona radiata L	689 (14.92)	411 (8.90)	34 (0.49)	0
Posterior corona radiata L^*^	133 (9.58)	0	157 (4.17)	324 (8.60)
Posterior thalamic radiation L^*^	447 (49.34)	54 (5.96)	149 (4.39)	347 (10.23)
Superior longitudinal fasciculus L	0	105 (19.85)	64 (3.13)	0

* *Regions where the size of significant AIL clusters decreased while the size of significant WMH clusters increased from baseline to 1 year after stroke. L, left*.

## Discussion

In this study, we developed a multiple-lesion symptom mapping approach based on support vector regression (SVR-MLSM), which considers both the presence of AIL and WMH, to investigate the strategic WMH locations for post-stroke cognitive impairment on a voxelwise basis. The strategic WMH locations identified by SVR-MLSM improved the prediction accuracy of the cognitive impairment both at baseline and at 1 year after stroke, compared with models that only used AIL locations as predictors. The findings of this pilot study confirmed the relevance of WMH location for post-stroke cognition and provided a more comprehensive map of strategic brain regions affected by acute event (AIL) and chronic event (WMH) for post-stroke impairment in global cognitive functioning.

To our knowledge, it was the first study that applied voxel-based analyses to explore the spatial relationships between two kinds of ischemic lesions (AIL and WMH) and post-stroke outcomes. Compared to mass-univariate VLSM and SVR-LSM which only consider the presence of a single type of lesion, SVR-MLSM tries to model more comprehensive information of the lesion status in a LSM problem where multiple kinds of lesions are presented. In detail, we represented each voxel in the lesion map with two lesion-specific variables which measured the lesion status of AIL and WMH respectively (Figure 1), and thus three lesion statuses (normal tissue or damaged with AIL or WMH) could be represented with these two variables. While the performance of the map-based methods SVR-LSM and SVR-MLSM can be measured through cross-validations with the optimized models from parameter training, it is not applicable for the mass-univariate VLSM. To make an equivalent comparison of the three LSM methods, we resorted to the significant anatomical regions (SVOI) identified by these methods and measured their contribution to behavior prediction. In general, the SVOI-AIL of SVR-LSM models had slightly better prediction performance than that of mass-univariate VLSM, which was in line with the reported merits of SVR-LSM over mass-univariate VLSM (Zhang et al., 2014). Furthermore, when the presence of WMH was also considered, the SVOI-AIL and SVOI-WMH generated from SVR-MLSM models jointly contributed more to the behavior prediction than the SVOI-AIL from the other models. These results verified our hypothesis about the additional impact of specific WMH locations on post-stroke cognition. As most of the included patients had small infarcts, it appeared that the MLSM models with or without SVOI-AIL as predictor had similar performance in behavior prediction (Table 3). Therefore, our results had less relevance with a cohort with more major stroke and might not be generalizable to patients with larger infarcts.

Of note, the SVR-MLSM models performed no better or even worse than the SVR-LSM models during parameter training prior to statistical inference (Table 2). This might result from the large number of noisy WMH voxels included in SVR-MLSM compared to SVR-LSM, as WMH is a chronic and relatively inferior event compared to AIL. The increased number of predictors with very limited contributions to the behavior being measured will inevitably cause lower performance during model training. In fact, the number of lesion-specific voxels for WMH (*N”* = 99,987) was much larger than that for AIL (*N'* = 39,051) in the LSM analyses. Although support vector regression can penalize the features that are irrelevant with the dependent variable, the prediction performance will be inevitably influenced by the curse of dimensionality if the number of noisy features is very large, and that's why denoising process like feature selection is often recommended (Weston et al., 2001; Guyon and Elisseeff, 2003). In a recent study, we demonstrated that feature selection could also improve the performance of SVR-LSM analyses where only the presence of AIL was considered (Zhao et al., 2017). In this study, we turned to the statistical inference which served as a post-denoising process rather than feature selection prior to model training, as feature selection for a lesion map with multiple lesion status in SVR-MLSM is more complex than that in SVR-LSM. In the subsequent comparison of the behavior prediction power based on statistical inference results (SVOI), the superior performance of SVR-MLSM over the other models demonstrated the effectiveness of this post-denoising procedure for the MLSM analyses.

Volume control is an issue commonly mentioned for lesion-symptom mapping. In this study, we applied different volume control strategies in the mass-univariate VLSM, SVR-LSM and SVR-MLSM models for a comprehensive comparison. Regarding the mass-univariate SVR-LSM models, the model without volume control (Model 4) performed better than that with volume control (Model 5) in behavior prediction based on SVOI-AIL (Table 3). This result coincided with the comment that regressing out the lesion size from behavior score might reduce the statistical power of LSM analyses (Karnath et al., 2004). Regarding the SVR-LSM models, the best volume control strategy (with the best prediction performance of SVOI-AIL in Table 3) was generally not consistent between the outcomes at baseline and 1 year after stroke. It indicated that the optimal volume control strategy for SVR-LSM might vary in different lesion-symptom mapping problems. Even though the nonlinear model with voxelwise normalization (Model 9) was proposed as the optimal approach in the published SVR-LSM paper (Zhang et al., 2014), it did no better than the linear model with voxelwise volume control (Model 7) in SVOI-based predictions (Table 3). This discrepancy might result from the relatively smaller lesion size and the insufficient lesion coverage of the brain in our study. Regarding the SVR-MLSM models, volume control by voxelwise normalization achieved the best performance in the SVOI-based predictions of both baseline and year 1 post-stroke outcomes (Table 3). However, the volume control method for SVR-MLSM should still be selected with caution when applied to other lesion-behavior data, as there might not be one-size-fits-all volume control strategy for SVR-MLSM just like the case of SVR-LSM as aforementioned.

The study cohort was carefully selected regarding the rough lesion sites of AIL. Only the patients with unilateral AILs in the left cerebral were included, where the associations between the AILs and post-stroke cognitive impairment (both short-term and long-term) were relatively stronger than those in the other lesion sites (infratentorial regions or right cerebral) (Hochstenbach et al., 2003; Bastos Leite et al., 2006). Even in this screened cohort with relatively important AIL sites, we identified WMH in the corpus callosum, corona radiata, and posterior thalamic radiation in both hemispheres as strategic substrates for short-term and long-term post-stroke cognitive impairment independent of AIL locations (Figure 6, Table 4). In fact, many of these white matter regions were shared by the significant clusters of AIL and WMH in our study (Table 5) and they corroborated with the strategic AIL locations identified in previous studies (Munsch et al., 2016; Biesbroek et al., 2017; Shahid et al., 2017; Zhao et al., 2017). Previous findings about the strategic WMH locations for post-stroke cognitive impairment were not consistent (Prins et al., 2004; Jokinen et al., 2005; Kandiah et al., 2011; Kang et al., 2013), where they only roughly divided WMH into perivascular WMH (PWMH) and deep WMH (DWMH). In our study, the identified significant WMH clusters covered both PWMH and DWMH for short-term and long-term outcomes. By comparing the significant lesion locations at baseline and 1 year after stroke, we found that AILs in the basal ganglia had a stronger long-term effect on the cognitive impairment (Figure 6, Table S2). In addition, as AILs became completely chronic lesions at 1 year after stroke, the influence of AIL decreased while the impact of preexisting WMH increased on post-stroke cognitive impairment in some specific regions, such as posterior thalamic radiation (Table 5). Such information is of clinical value, as this provides hints to understand the long-term effect of AIL and preexisting WMH and the alteration of their roles with time in post-stroke cognitive impairment.

There are several limitations to this study that should be taken into account. Firstly, the sample size of the study cohort is relatively small and the lesion distribution was relatively focal without sufficient lesion coverage (especially for AIL) throughout the brain, as we applied very strict exclusion criteria to remove potential confounders of the multiple-lesion symptom problem. In fact, only the patients with relatively mild to moderate stroke can be included in this study, because it would be difficult for the patients with severe stroke to accomplish MoCA assessments (Chiti and Pantoni, 2014), and that only including stroke patients with MRI may be systematically biased toward smaller strokes (Sperber and Karnath, 2017). As only a limited portion of the brain was damaged with AIL in our cohort, the power of the MLSM analyses might be influenced and the results regarding the identified strategic WMH locations on top of AIL should be explained with caution. Moreover, the significant clusters of AIL in our study was largely based on only three to five patients (Figure 7), and the independent contribution of these clusters to the behavior being measured was only slight compared with WMH clusters (by comparing Model 16~18 with Model 13~15 in Table 3). This might further limit the statistical power of our results, which also requires a larger cohort with sufficient lesion coverage of AIL in the brain to validate our findings in the future. Secondly, we did not test the prediction effect of the statistical results (SVOI-AIL and SVOI-WMH) from the LSM analyses on an external validation set due to our small sample size. Consequently, some of our findings may not be generalizable to the patients with significantly different lesion distribution. Thirdly, we tested the performance of different volume control strategies on the real lesion-behavior data, although a more favorable way is to compare their performance on a simulation dataset based on the lesion data (Zhang et al., 2014; Sperber and Karnath, 2017). However, as two kinds of lesions are considered in the SVR-MLSM at the same time, the volume control is more complex as it involves the lesion size of both lesions. As the distribution of AIL is relatively focal to a limited portion of the brain, it's also difficult to select specific regions of interest with sufficient lesion prevalence of both AIL and WMH to construct the simulation as in the SVR-LSM paper (Zhang et al., 2014). Finally, the long-term cognitive function was assessed at 15–18 months after stroke, and there might be some change in the brain compared to baseline MRI for the included patients. Therefore, the MLSM map for the long-term outcome should be interpreted with caution. However, we excluded the patients who had incident stroke at 15–18 months, as the hospital would perform rescanning (at least with a CT scan) for the patients with recurrent stroke to describe the type and location of stroke. This additional exclusion criterion helped to make the lesion map at follow-up close to the baseline as much as possible. The analysis of the impact of WMH progression, which is another potential confounder that mediates the influence of baseline WMH locations on the long-term post-stroke outcomes (Prins and Scheltens, 2015), is currently not available in this study, as most of the patients without recurrent stroke would not be rescanned at follow-up due to the general hospital routine. In this case, the impact of WMH progression can only be evaluated in the future studies where follow-up brain MRI scans are available for all the included patients.

In conclusion, this study provides a novel perspective to measure the additional contribution of WMH locations to post-stroke cognitive impairment, using the proposed multiple-lesion symptom mapping approach on a voxelwise basis. The maps of significant brain regions affected by AIL and WMH for short-term and long-term global cognitive impairment may help clinicians to understand the cognitive impact of AIL and WMH locations, and initiate adequate rehabilitation strategies for the ischemic stroke patients at the earliest possible stage. The multiple-lesion symptom mapping approach can also be applied to other cohorts where multiple kinds of lesions often coincide in the brain and have potential contributions to a specific behavior.

## Author contributions

LZ: theoretical development, data analysis, manuscript preparation. AW: clinical advice. YL: image registration. WL: lesion delineation. WC: data collection. JA: data collection. RL: data collection. VM: clinical advice and study design. LS: study design, commenting on drafts.

### Conflict of interest statement

The authors declare that the research was conducted in the absence of any commercial or financial relationships that could be construed as a potential conflict of interest.
