# Supplementary material for: The Additional Contribution of White Matter Hyperintensity Location to Post-stroke Cognitive Impairment: Insights From a Multiple-Lesion Symptom Mapping Study
Source: Front Neurosci. 2018 May 1;12:290. doi: 10.3389/fnins.2018.00290 (PMC5938410; doi:10.3389/fnins.2018.00290)
Supplement: Supplementary file 1 [file Data_Sheet_1.docx]

# SUPPLEMENTAL INFORMATION

## Supplementary figure


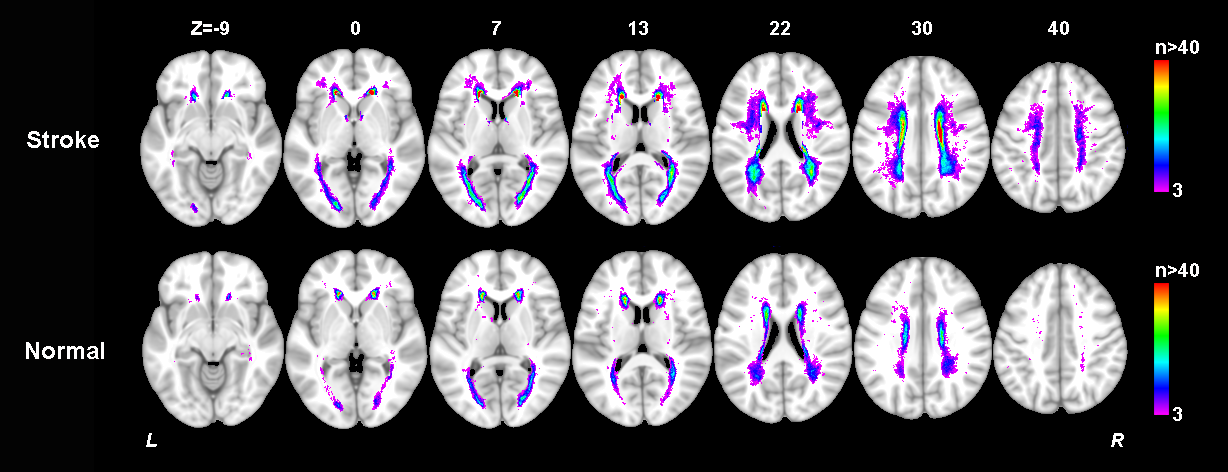


**Figure S1**. Lesion prevalence of while matter hyperintensity in the stroke cohort of our study and a normal control cohort well-matched in age, gender and education. Voxels that are damaged in at least three patients are projected on the 1mm MNI-152 template (Z coordinates: -9, 0, 7, 13, 22, 30, 40). Bar indicates the number of patients with a lesion for each voxel.


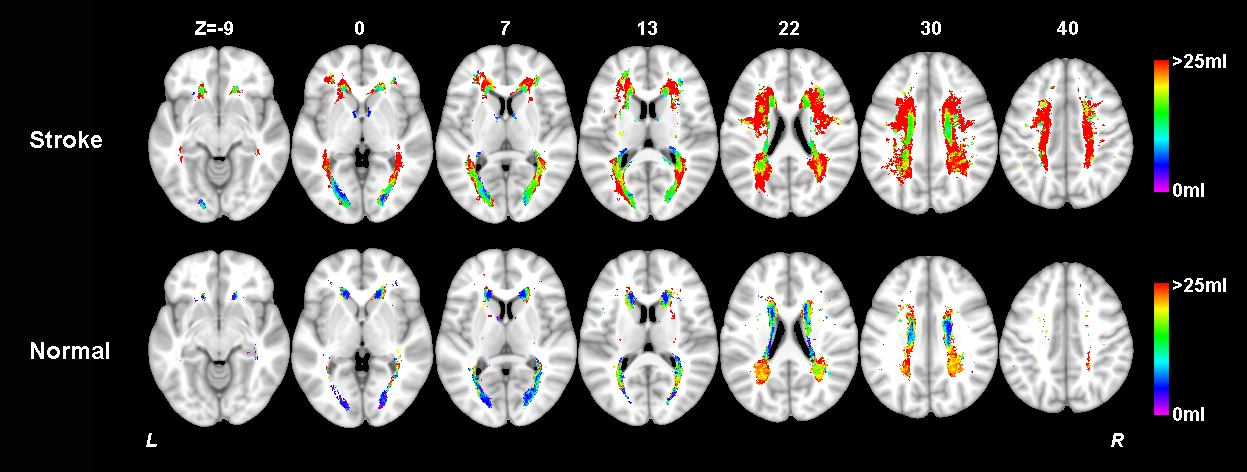


**Figure S2**. Lesion size topologies of while matter hyperintensity in the stroke cohort of our study and a normal control cohort well-matched in age, gender and education. Bar indicates the median white matter hyperintensity volume a patient would have, given that the specific voxel is lesioned.


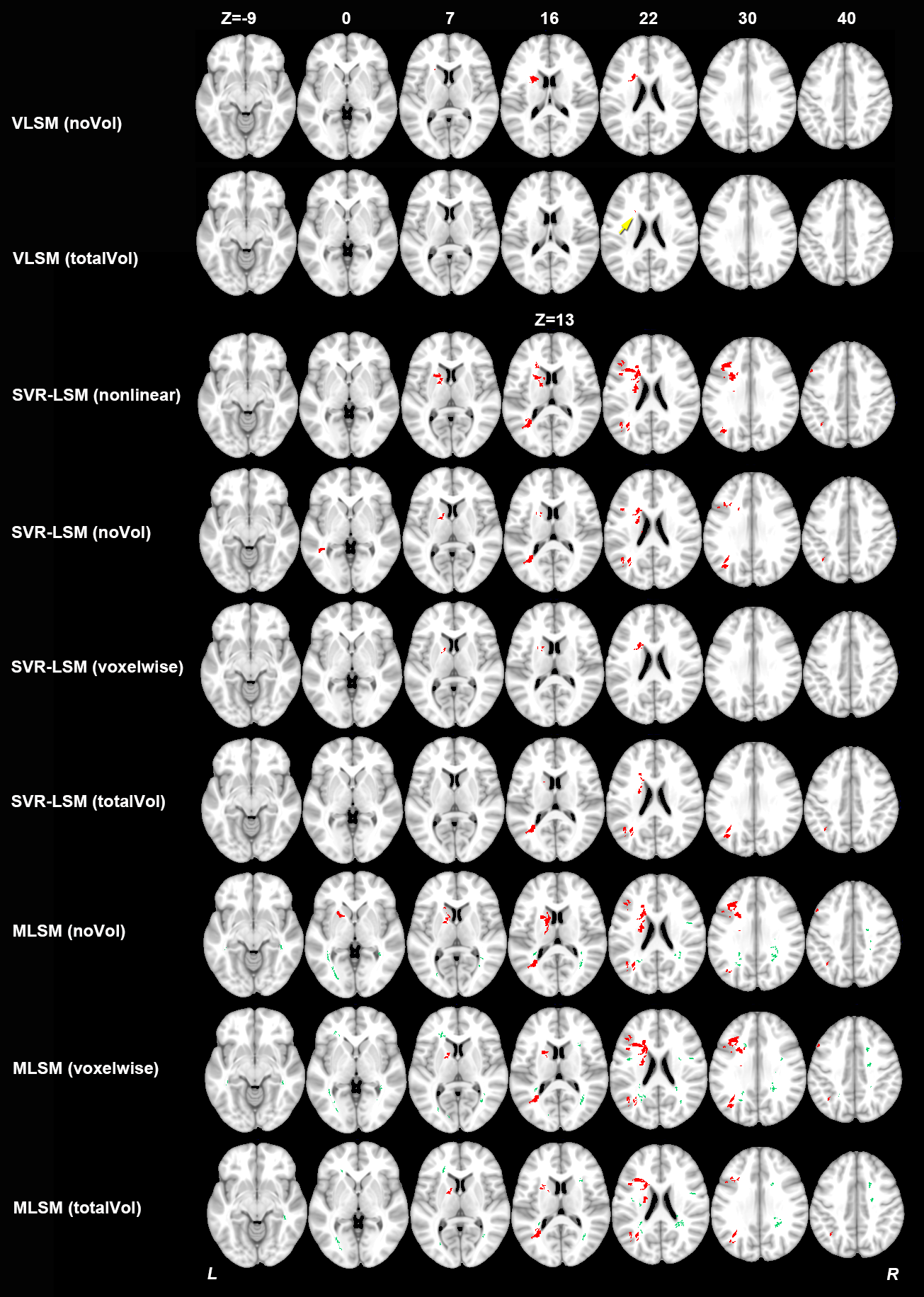


**Figure S3**. Statistical inference results of the mass-univariate VLSM, SVR-LSM and SVR-MLSM models for baseline cognitive impairment. The significant clusters of AIL (in red) and WMH (in green) were shown with p<0.05 from statistical inference with 1000 permutations. The yellow arrow points to the small significant cluster of VLSM results. noVol: without volume control; voxelwise: voxelwise normalization by weighting each voxel with inverse proportion to the square root of the corresponding lesion size: totalVol, volume control by regressing out the total lesion burden for baseline MoCA.


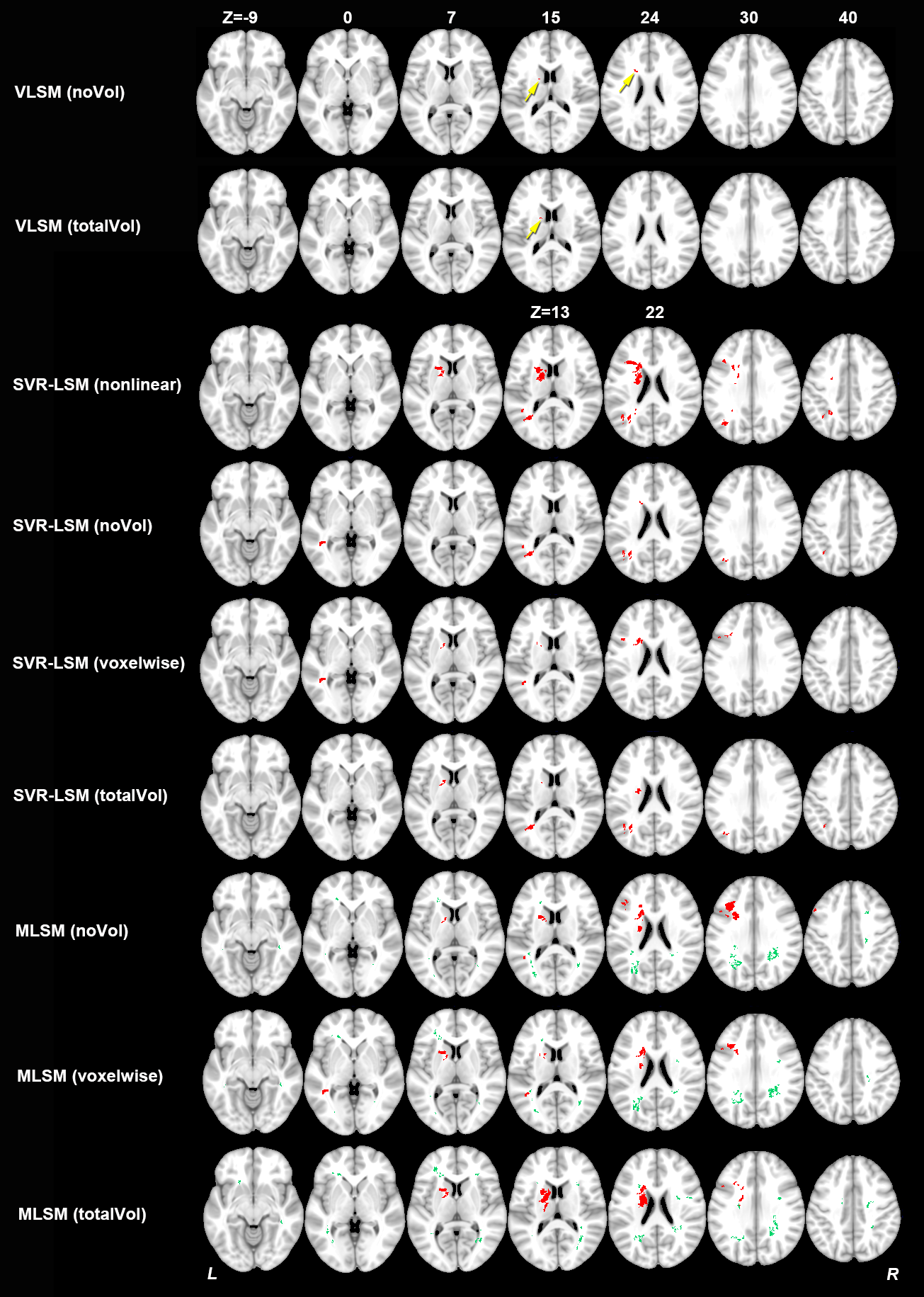


**Figure S4**. Statistical inference results of the mass-univariate VLSM, SVR-LSM and SVR-MLSM models for cognitive impairment at one year after stroke. The significant clusters of AIL (in red) and WMH (in green) were shown with p<0.05 from statistical inference with 1000 permutations. The yellow arrow points to the small significant cluster of VLSM results. noVol: without volume control; voxelwise: voxelwise normalization by weighting each voxel with inverse proportion to the square root of the corresponding lesion size: totalVol, volume control by regressing out the total lesion burden for year 1 MoCA.


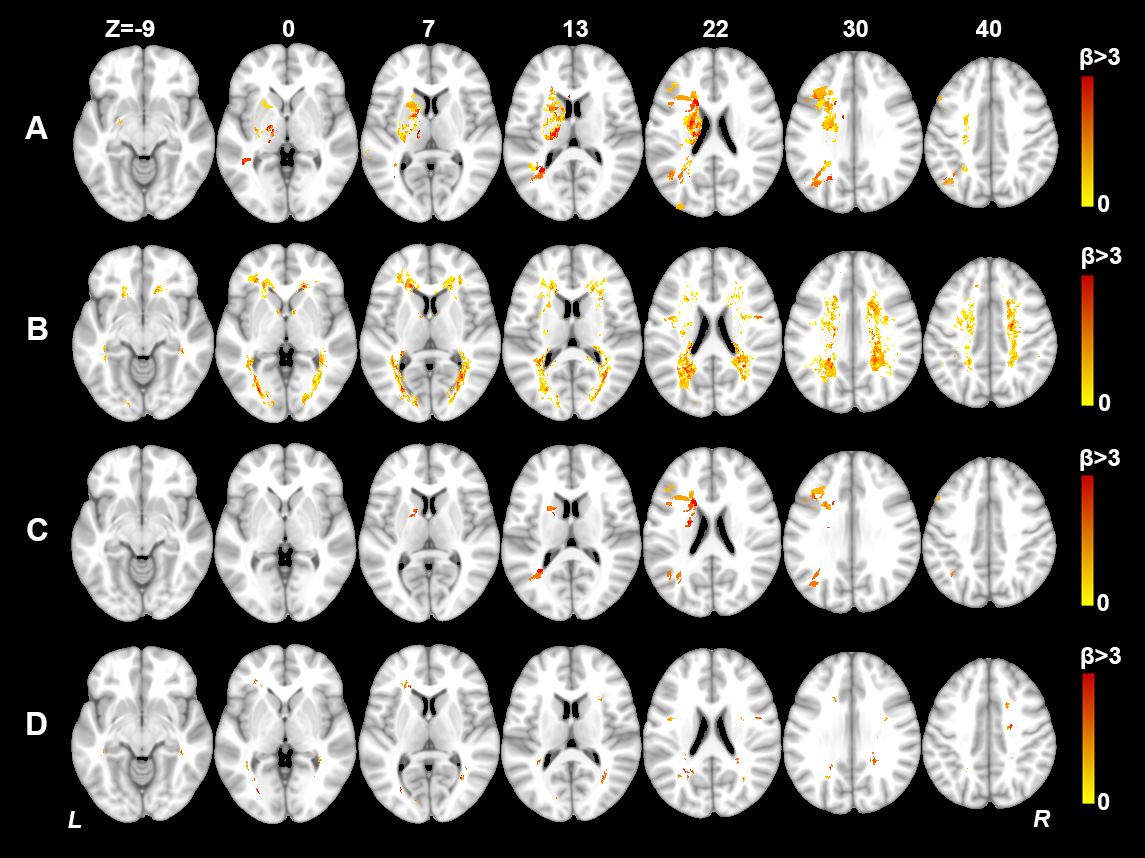


**Figure S5**. Beta map of SVR-MLSM (with volume control by voxelwise normalization) for baseline cognitive impairment. (A) Non-thresholded beta map of AIL; (B) Non-thresholded beta map of WMH; (C) Thresholded beta map of AIL with p<0.05 in the 1000 permutations; (D) Thresholded beta map of WMH with p<0.05 in the 1000 permutations. The beta maps of the AIL and WMH of the SVR-MLSM were normalized together to have a standard deviation of 1 for better visualization and comparison. Only the voxels with positive betas are displayed here.


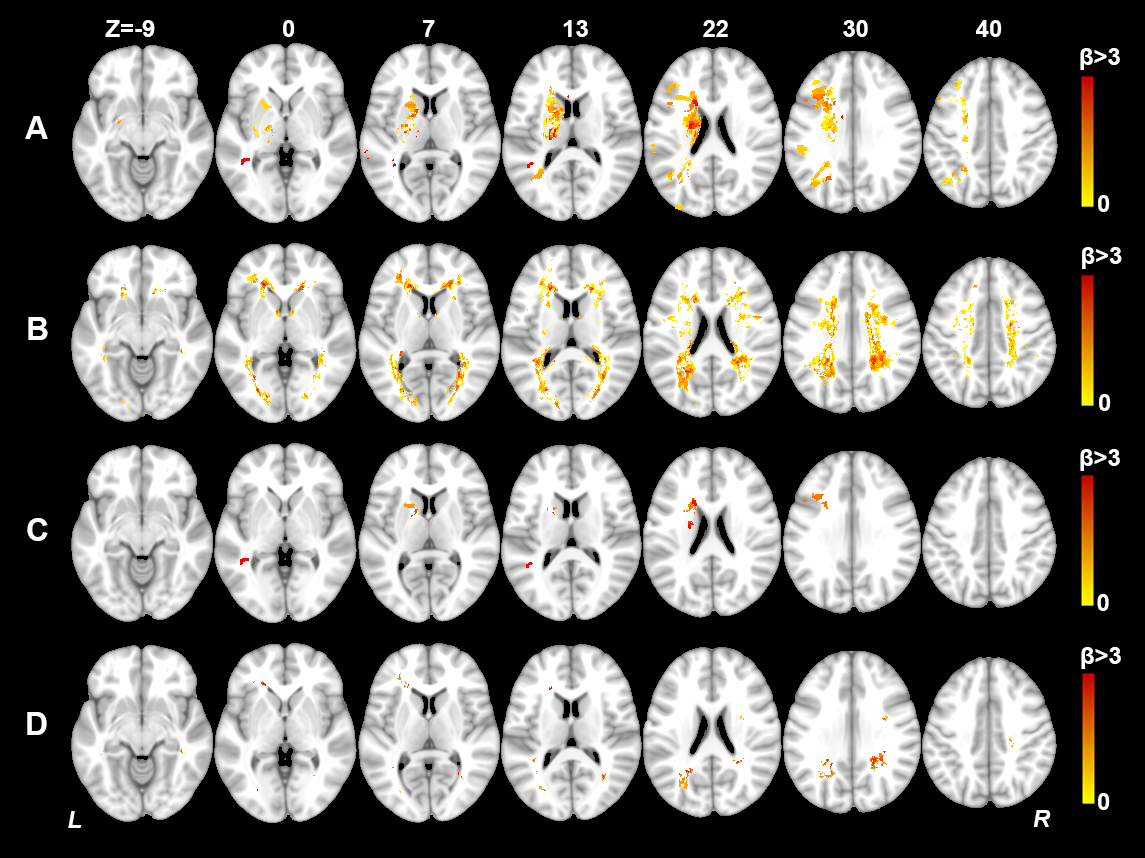


**Figure S6**. Beta map of SVR-MLSM (with volume control by voxelwise normalization) for cognitive impairment at one year after stroke. (A) Non-thresholded beta map of AIL; (B) Non-thresholded beta map of WMH; (C) Thresholded beta map of AIL with p<0.05 in the 1000 permutations; (D) Thresholded beta map of WMH with p<0.05 in the 1000 permutations. The beta maps of the AIL and WMH of the SVR-MLSM were normalized together to have a standard deviation of 1 for better visualization and comparison. Only the voxels with positive betas are displayed here.

## Supplementary table

**Table S1**. Characteristics of the subjects in our stroke cohort and normal control cohort

| **Characteristics** | **Stroke cohort (n=76)** | **Normal controls (n=76)** | ***p-value**** |
| --- | --- | --- | --- |
| Age, mean ± SD (years) | 65.8 ± 10.1 | 68.0 ± 6.1 | 0.105 |
| Education, mean ± SD (years) | 6.8 ± 4.2 | 6.8 ± 3.8 | 0.984 |
| Female, n (%) | 32 (42.1) | 36 (47.4) | 0.625 |
| WMH volume, mean ± SD (ml) | 14.2 ± 13.4 | 5.9 ± 8.4 | 1.30E-05 |
| Baseline MoCA, mean ± SD | 21.6 ± 5.7 | 23.6 ± 3.2 | 0.009 |

*Group comparison were performed between the characteristics of the two groups with independent t-test or chi-square test where appropriate.

**Table S2**. SVR-MLSM results of acute ischemic lesions

| Region | Patients with lesion (n)* | Region size in voxel (n) | Tested voxels (n) | Significant voxels of AIL [n (%)] | |
| --- | --- | --- | --- | --- | --- |
|  |  |  |  | Baseline MoCA | Year 1 MoCA |
| Middle frontal gyrus L | 5 | 36072 | 2569 | 859 (33.44) | 182 (7.08) |
| Inferior frontal gyrus (opercular) L | 5 | 8304 | 385 | 81 (21.04) | 38 (9.87) |
| Inferior frontal gyrus (triangular) L | 5 | 20232 | 955 | 741 (77.59) | 98 (10.26) |
| Middle temporal gyrus L | 4 | 39536 | 486 | 129 (26.54) | 50 (10.29) |
| Middle occipital lobe L | 4 | 26160 | 1223 | 256 (20.93) | 0 |
| Inferior parietal gyrus L | 5 | 19576 | 1513 | 26 (1.72) | 0 |
| Angular L | 4 | 9384 | 1044 | 399 (38.22) | 0 |
| Caudate L | 19 | 7696 | 2404 | 338 (14.06) | 342 (14.23) |
| Putamen L | 17 | 8072 | 3487 | 0 | 136 (3.90) |
| Pallidum L | 10 | 2344 | 816 | 39 (4.78) | 12 (1.47) |
| Body of corpus callosum | 5 | 17849 | 449 | 60 (13.36) | 36 (8.02) |
| Anterior limb of internal capsule L | 14 | 2889 | 1761 | 745 (42.31) | 649 (36.85) |
| Anterior corona radiata L | 6 | 7507 | 747 | 413 (55.29) | 0 |
| Superior corona radiata L | 17 | 8929 | 4619 | 689 (14.92) | 411 (8.90) |
| Posterior corona radiata L | 11 | 5325 | 1388 | 133 (9.58) | 0 |
| Posterior thalamic radiation L | 4 | 6387 | 906 | 447 (49.34) | 54 (5.96) |
| External capsule L | 12 | 3909 | 566 | 54 (9.54) | 0 |
| Superior longitudinal fasciculus L | 4 | 9386 | 529 | 0 | 105 (19.85) |
| Superior fronto-occipital fasciculus L | 13 | 499 | 390 | 254 (65.13) | 179 (45.90) |

Regions where there were significant AIL clusters (p<0.05) for global cognitive impairment at baseline and one year after stroke. The remaining regions in AAL atlas or ICBM-DTI-81 white matter tract atlas contained no significant voxels for cognitive impairment either at baseline or at one year after stroke; these regions are not shown here. L, left. * Number among 76 included patients had an AIL that overlapped (≥1 voxel) with the specified region of interest in AAL atlas and ICBM-DTI-81 white matter tract atlas.
